# The Clinical Impact of the Extension of Acute Type A Aortic Surgery on Long-Term Outcomes: Should We Tend to Be Conservative?

**DOI:** 10.3390/medicina60010035

**Published:** 2023-12-24

**Authors:** Natasa Jankovic, Milos Matkovic, Ilija Bilbija, Vladimir Milicevic, Mina Zlatkovic, Nemanja Aleksic, Vladimir Cvetic, Jelena Milin-Lazovic, Svetozar Putnik

**Affiliations:** 1 Department for Cardiac Surgery, University Clinical Centre of Serbia, 11000 Belgrade, Serbia; dr.matko@hotmail.com (M.M.); i.bilbija@yahoo.com (I.B.); vladodrillwork@gmail.com (V.M.); zlatkovic.mina@gmail.com (M.Z.); ner.vuk@hotmail.com (N.A.); svetozar073@yahoo.com (S.P.); 2 Faculty of Medicine, University of Belgrade, 11000 Belgrade, Serbia; drvladimircvetic@gmail.com (V.C.); milinjelena@gmail.com (J.M.-L.); 3 Department for Cardiovascular Radiology, University Clinical Centre of Serbia, 11000 Belgrade, Serbia; 4 Department of Biostatistics, Faculty of Medicine, University of Belgrade, 11000 Belgrade, Serbia

**Keywords:** aortic dissection type A surgery, aortic arch surgery, outcome, quality of life

## Abstract

*Background and Objectives*: Despite advances in surgical techniques, industry adjuncts, and cerebral perfusion techniques, the in-hospital mortality rate of type A acute dissection (TAAD) remains at 15–30%. This study aimed to investigate the influence of different extents of aortic resection on survival and quality of life (QoL) after long-term follow-up. *Materials and Methods*: A retrospective observational trial was performed, including 165 patients operated upon for TAAD. Patients were divided into two groups according to the extent of their aortic repair: the first group comprised patients who had ascending aorta replacement and the second included patients who had hemiarch or total arch replacement. The groups were compared with regard to their baseline characteristics, operative characteristics, survival, complications, and QoL during nine years of follow-up. *Results*: The mean follow-up time was 75.6 months (1–108 months). The mean survival in the ascending aorta repair group was 89.651 (81.242–98.061) months and was 54.801 (40.053–69.548) months in the hemiarch and arch group; the difference between the groups was significant (log-rank *p* < 0.001). The rate of new postoperative neurological deficits was statistically higher in the hemiarch and arch group (17.5% vs. 8.4%, *p* = 0.045), the most common being stroke, and was also more frequent in the hemiarch and arch group than in the ascending aorta group (with statistical significance (15.7% vs. 6.5%)). The mean SF-12 physical score from the QoL questionnaire was higher in the ascending aorta replacement group than in the hemiarch and arch group (50.1 ± 7.3 vs. 44.0 ± 11.9, *p* = 0.017). Additionally, the mean SF-12 mental score was higher in the ascending aorta replacement group (52.3 ± 7.3 vs. 47.1 ± 12.8, *p* = 0.032). *Conclusions*: A more aggressive approach involving aortic arch repair means a lower survival rate and lesser quality of life after long-term follow-up in comparison with the replacement of the ascending aorta. If clinically applicable, a more defensive strategy may be considered.

## 1. Introduction

Cardiac surgeons worldwide continue to evolve their surgical techniques in order to reduce the in-hospital mortality of type A acute dissection (TAAD) to below a two-digit number. Despite advances in surgical techniques, industry adjuncts, and cerebral perfusion techniques, in-hospital mortality remains at 15–30% [1,2]. The large variations in outcome are attributed to the extent of the dissection itself, the location of the intimal tear, the presence of distal malperfusion, delayed clinical diagnosis, comorbidities, and age. Finally, the experience of the surgeon and the centre in aortic surgery plays an important role [3].

The safest and most conservative approach is the excision of the tear and replacement of the ascending aorta. Thus, the complex replacement of the dissected arch is avoided, and the operative risk is mitigated. However, the residual patent false lumen in the aortic arch poses a risk of aneurysmal dilatation in this section, which then magnifies morbidity and mortality and leads to the subsequent need for reoperation [4,5].

In recent years, the frozen elephant trunk technique (FET) has evolved in everyday clinical practice as well as in emergency settings [6]. In case of involvement of the aortic arch and distal descending aorta, it can be used in the treatment of TAAD. In this case, the procedure is a single-stage instead of two-staged, as initially intended. The stent is deployed in the descending aorta in an attempt to close the false lumen and promote thrombosis. As this is a complex procedure, it is currently reserved for this type of TAAD treated in high volume FET centres [7].

The prevention of death from TAAD rupture is mainly accomplished via the excision of proximal entry tears, as stated by The International Registry for Aortic Dissection (IRAD) [8]. Nevertheless, the reduction in operative risk through avoiding arch surgery in this setting comes with the price of later reintervention, making the decision to extend the procedure very challenging [9]. The decision of the extension of the procedure is not straightforward. Aside from the choice between total arch and isolated ascending aorta replacement, there are many variations in the surgical approach regarding composite root surgery, open or frozen elephant trunk, and descending thoracic aorta procedures. The absence of randomised trials, large variations in clinical variables, and different surgeon and centre experiences make a general agreement or recommendation (limited versus extended) very difficult to achieve.

This study aimed to investigate the influence of different extents of aortic resection on survival and quality of life (QoL) after long-term follow-up.

## 2. Materials and Methods

### 2.1. Study Design

A retrospective observational study was performed. The ethical committee approved the study (ECCS-2021/56), and informed consent for participation in the study was obtained from all patients.

**Figure 1:**
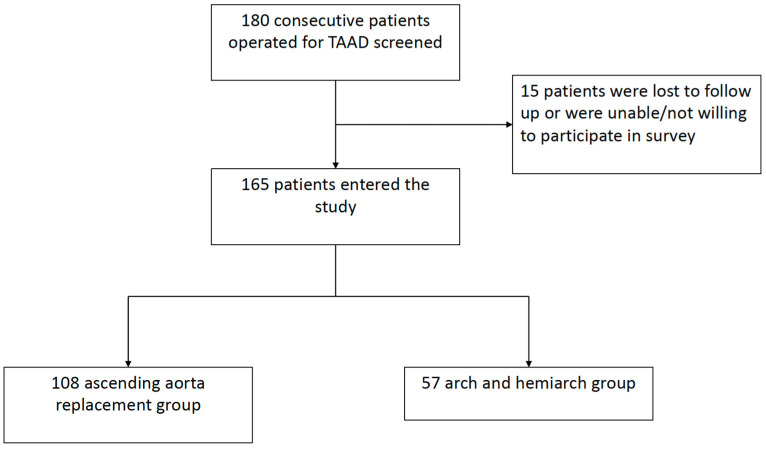
Flowchart of patient enrolment in the study. TAAD: type A acute aortic dissection.

Between January 2012 and December 2016, 180 consecutive patients underwent urgent surgery for acute Stanford type A acute aortic dissection at the University Clinical Center of Serbia. This observational analysis included patients operated upon for acute TAAD; chronic TAAD and iatrogenic aortic dissection were exclusion criteria. Ten patients were lost to follow-up, while five patients refused to participate in the research (Figure 1).

Patients were divided into two groups according to the extent of their aortic repair: the first one comprised patients who had ascending aorta replacement and the second included patients who had hemiarch or total arch replacement. The groups were compared with regard to their baseline characteristics, operative characteristics, survival, complications, and QoL during nine years of follow-up. The data were obtained from medical records and via telephone surveys during the follow-up period.

### 2.2. Surgical Procedure

The patient was admitted within the aortic centre protocol of our institution. A computed tomography (CT) scan was performed on admission, and aortic dissection was diagnosed according to definitions from the current guidelines [10]. Immediately afterwards, two arterial and two venous lines were placed as well as a central venous line. After the induction of anaesthesia, transoesophageal echocardiography was performed to evaluate the competency of the aortic valve.

The approach was carried out through median sternotomy.

The arterial cannulation was performed through the right axillary artery, brachiocephalic trunk, or femoral artery. The brachiocephalic trunk was cannulated using an 8 mm Dacron graft using a 5-0 Prolene suture and a side clamp. If the right axillary artery was used, an 8 mm Dacron graft was anastomosed to the vessel with 5-0 Prolene and, if necessary, a 19 or 21 Fr cannula was inserted into the graft and secured. The femoral artery was cannulated with a 19–23 French arterial cannula using the Seldinger technique, or directly with the opening of the vessel, which was closed with 6-0 Prolene after decannulation. Cold crystalloid St. Thomas cardioplegia was used to obtain cardioplegic arrest. A bolus of unfractionated heparin of 300–400 IU per kg body weight was administered prior to cannulation and establishment of a cardiopulmonary bypass (CPB) to achieve an activated clotting time of more than 460 s. After the initiation of systemic cooling on the CPB, a left ventricle vent catheter was usually placed through the right superior pulmonary vein. After the induction, a cardiopulmonary bypass (CPB) aortic cross-clamp was applied while proximal anastomosis on the aorta was performed, followed by open distal anastomosis in circulatory arrest. In exceptional cases, a distal-first technique was used. Moderate (24–28 C) and deep hypothermia (18 C) were used alongside anterior cerebral perfusion (ACP), if applicable. The anterograde cerebral perfusion was performed via the right axillary artery or brachiocephalic trunk or with separate self-inflatable catheters placed into the supra-aortic branches in case of femoral cannulation. The CPB flow during ACP was 10 mL/kg. If the Bental procedure was performed, the composite graft with mechanical aortic valve prosthesis was used. In the case of aortic arch reconstruction, a Dacron tubular graft was used if the Carrel patch technique was used for aortic branches reimplantation, or a branched graft (JOTEC GmbH, Hechingen, Germany) was used if they were reimplanted separately.

According to local institutional protocol, the primary goal was the exclusion of the primary intimal tear. The extent of aortic repair was decided by the surgical team based on CT and in situ operative findings. The decision to proceed with the aortic arch reconstruction was made if the intimal tear was found in that zone. Also, this approach was favoured if the dissection gravely affected this zone or if the supra-aortic branches, as well as the aortic arch, were dilated over 5 cm. Advanced patient age and serious comorbidities, alongside a high frailty score, were also taken into account when considering the extent of the surgery.

### 2.3. Definitions and Study Endpoints

The data were extracted from our institutional registry, a prospectively maintained clinical registry of all patients undergoing aortic dissection surgery at our institution, and double-checked for accuracy. All operative survivors were followed up regularly, and follow-up was completed by all 165 patients (100%). All clinically gathered data, including adverse events during follow-up and cause of death, were registered and reported according to the standardised institutional protocol. Adverse events included postoperative events and events during follow-up. The postoperative events recorded were re-exploration for bleeding, acute kidney failure, need for haemodialysis, new neurological events, pneumonia, sepsis, wound infections, lower limb ischemia, gastrointestinal bleeding, and in-hospital mortality. In the follow-up need for reoperation, new cardiovascular events and new strokes were recorded.

### 2.4. Quality of Life Survey

QoL was estimated using the QoL Short Form Survey (SF-12). The SF-12 is derived from the SF-36 Short Form survey and scores the responses of the mental and physical components of the study. The physical SF-12 component investigates physical functioning, pain, and roles, while the cognitive component summarises individuals’ mental health and social and emotional functioning. The results of these two components were scored from 0 to 100 using standard answers to standard questions, wherein higher scores represented better mental and physical health. The SF-12 quality-of-life questionnaire consists of twelve questions that measure eight health domains to assess physical and mental health. The physical health-related domains include general health (GH), physical functioning [11], role physical (RP), and body pain (BP). The mental health-related scales include vitality (VT), social functioning (SF), role emotional (RE), and mental health (MH). The questionnaire has been validated across a number of chronic diseases and conditions. We administered the SF-12v2^®^ by telephone survey to study participants. For each participant, we then calculated two summary scores of the SF-12v2^®^—physical and mental health—using the weighted means of the eight domains.

### 2.5. Statistical Analyses

Descriptive statistics were calculated for the baseline demographic and clinical features and treatment outcomes. Graphical and mathematical methods tested the normality of distribution. As appropriate, continuous variables were presented as means with standard deviations or medians with 25th–75th percentiles. Categorical variables were presented as numbers and percentages. Differences between groups were analysed using the Student’s *t*-test for continuous variables (or the Mann–Whitney test) and the Pearson chi-squared test for categorical variables. Survival analysis was performed using the Kaplan–Meier method, and the groups were compared using the log-rank test. In addition, the Kaplan–Meier survival curves were truncated at a timepoint in follow-up when at least 10% of patients were still at risk, to avoid visual misinterpretation [12]. The significance level was set at 0.05, and all testing was two-sided. Statistical analysis was performed using IBM SPSS Statistics for Windows, version 21.0. (Armonk, NY, USA).

## 3. Results

### 3.1. Demographic and Preoperative Data

There were fewer patients in the hemiarch and arch group than in the ascending aorta group (*n* = 108 vs. *n* = 57). The baseline characteristics of both groups of patients are shown in Table 1. The groups did not differ statistically in demographic characteristics or comorbidities on admission. The frequency of male patients dominated both groups but there was no statistically significant difference between them (80.6% vs. 73.3%, *p* > 0.05). The rate of cardiogenic shock on admission, rate of cardiac tamponade, and pericardial effusion also did not differ between the groups.

### 3.2. Intraoperative Data

An analysis of the intraoperative parameters demonstrated a higher CPB time and duration of circulatory arrest in the arch and hemiarch group in comparison to the ascending aorta replacement group (241 ± 65 min vs. 175 ± 52 min, *p* = 0.013; 34 ± 20 vs. 20 ± 7, *p* = 0.015) (Table 2). The mean aortic cross-clamp time did not differ between the two groups (98 ± 51 vs. 89 ± 21, *p* = 0.532). In both groups, cerebral perfusion during circulatory arrest was used in over 70% of patients during surgery; no statistically significant difference was observed (79.6% vs. 77.2%, *p* = 0.638). The most frequent cannulation site was the brachiocephalic trunk, followed by the femoral artery and right axillary artery, without a statistically significant difference between the groups. Additionally, there was no statistically significant difference in the frequency of Bental procedures performed, or in the number of concomitant coronary artery bypass grafts in the hemiarch and arch group compared to the ascending aorta group.

### 3.3. Perioperative Outcomes

An analysis of the postoperative outcomes is shown in Table 3. The ascending aorta group had a trend towards more re-exploration for bleeding than the hemiarch and arch group; however, there was no statistically significant difference in these data (21.5% vs. 10.7%, *p* = 0.087). The most common postoperative complication was sepsis, which was evenly distributed between the groups (21.4% vs. 19.6%, *p* = 0.786). The rate of new postoperative neurological deficits was statistically higher in the hemiarch and arch group (17.5% vs. 8.4%, *p* = 0.045), with the leading cause being stroke, which was also more frequent in the hemiarch and arch group than in the ascending aorta group (with statistical significance (15.7% vs. 6.5%)). Additionally, the occurrence of coma was higher in the hemiarch and arch group, reaching a statistically significant value (7.1% vs. 2.8%, *p* = 0.014).

The rate of acute kidney failure, haemodialysis, pneumonia, wound infection, lower limb ischemia, and gastrointestinal bleeding did not differ between the groups after analysis. The in-hospital mortality was statistically higher in the arch group in comparison to the ascending aorta replacement group (28.1% vs. 18.5%, *p* = 0.035).

### 3.4. Long-Term Outcomes

**Figure 2:**
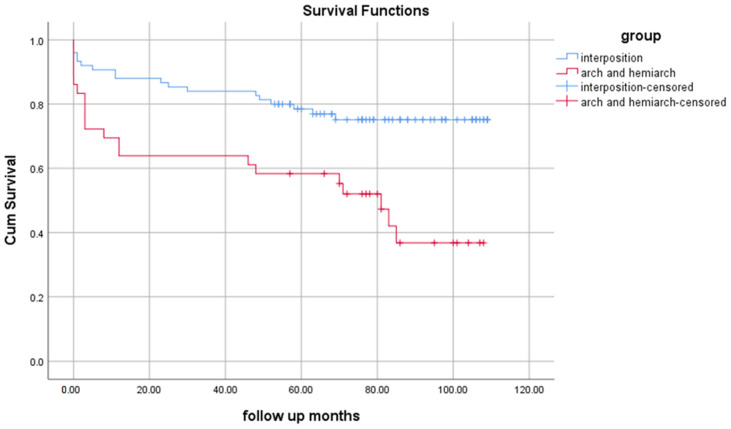
Cumulative survival in patients with hemiarch and arch replacement and ascending aorta replacement.

The mean follow-up was 75.6 months (1–108 months). The rate of reoperation and new neurological or cardiovascular events did not differ statistically upon follow-up (Table 3). The mean survival rate was 77.543 (69.303–85.603) months; 8 (4.8%) patients died during surgery and 36 (21.8%) died during hospitalisation. The mean survival in the ascending aorta repair group was 89.651 (81.242–98.061) months, and was 54.801 (40.053–69.548) months in the hemiarch and arch group; the difference between the groups was significant (log-rank test *p* < 0.001), Figure 2.

The predictors of mortality and their hazard ratios are reported in Table 4. The independent predictors of greater mortality were age (HR = 1.090, 95% CI = 1.052–1.131, *p* < 0.001), hemiarch and arch group (HR = 3.446, 95% CI = 1.848–6.426, *p* < 0.001), the presence of coronary disease (HR = 6.340, 95% CI = 2.810–14.095, *p* < 0.001), re-exploration for bleeding (HR = 2.2014, 95% CI = 1.011–4.807, *p* = 0.047), acute kidney injury (HR = 2.749, 95% CI = 1.153–6.152, *p* = 0.023), haemodialysis (HR = 4.530, 95% CI = 1.377–14.989, *p* = 0.013), stroke (HR = 3.590, 95% CI = 1.646–7.829, *p* = 0.001), coma (HR = 16.214, 95% CI = 5.252–50.058, *p* = <0.001), pneumonia (HR = 5–277, 95% CI = 2.054–13.5861, *p* < 0.001), and sepsis (HR = 2.046, 95% CI = 1.043–4.013, *p* = 0.037).

### 3.5. Quality of Life

SF-12 physical and mental scores were obtained from 135 patients (81.1%) during follow-up. The mean SF-12 physical score was 50.1 ± 7.3 in the ascending aorta group, while in the hemiarch and arch group, it was 44.0 ± 11.9, which was statistically significant (*p* = 0.017). Additionally, the mean SF-12 mental score was higher (52.3 ± 7.3) in the ascending aorta group in comparison to the hemiarch and arch group (47.1 ± 12.8), reaching statistical significance at *p* = 0.032.

## 4. Discussion

The present study aimed to analyse the impact of the extent of resection and replacement of the aorta during surgery for TAAD. The significant findings were as follows: (1) Resection procedures that included hemiarch or total arch replacement are an independent predictor of poor survival; (2) have lower in-hospital and long-term survival rates and greater incidence of postoperative neurological events; and (3) produce lower physical and mental QoL scores within nine years of follow up.

Acute aortic dissection remains an urgent condition, with its mortality rate still reaching up to 30% even in the most experienced centres [13]. The primary goal of dissection is to exclude intimal tears, which are usually located in the ascending aorta; however, the question of how much of the dissected arch should be resected then arises [14]. As the patent false lumen poses a risk of forming aneurysms in the arch, as well as a subsequent risk of rupture, several authors have suggested an aggressive initial approach to replace part of or the entire aortic arch [15,16]. However, this approach adds significantly to the complexity of surgery; thus, the optimal approach is still being debated [17]. Yang et al. conducted a study that included more than 400 patients operated upon for TAAD with either aggressive total arch replacement (*n* = 150) or a more conventional approach (*n* = 332) [14]. Their protocol was to exclude more than 4 cm of the aorta after the intimal tear, alongside aggressive resection of the supra-aortic branches if they are dissected. After a 15-year follow-up, they reported no significant difference in survival between the total arch and the conservative approach groups (log-rank *p* = 0.55, 10-year survival 70% vs. 72%). Additionally, they reported better in-hospital survival than IRAD, the German Registry for Acute Aortic Dissection type A (GERADAA), and the STS Database (7–9% in the study vs. 10–30% in the registries) [8,18,19]. The authors gave the following potential explanation for their success: their approach was to treat malperfusion syndrome with fenestration and stenting first, and then proceed with the surgery. However, in the present study, the in-hospital mortality in the arch group was higher than in the ascending aorta replacement group (28.1% vs. 18.5%, *p* = 0.035). Additionally, in our study, after nine years of follow-up, the Kaplan–Meier analysis demonstrated better survival in the ascending aorta group in comparison to the arch and hemiarch group, at 89.651 (81.242–98.061) months vs. 54.801 (40.053–69.548) months, log-rank *p* < 0.001. A study with a similar design to the present one conducted by Merkle et al. revealed findings that appear to reinforce our results [20]. Their study included 240 consecutive patients operated upon for TAAD. Their analysis assessed the outcomes of three groups divided according to the extent of their aortic resection: the ascending aorta group, the hemiarch group, and the total arch group. The total arch group had a significantly lower in-hospital and 30-day mortality in comparison to the more conventional approach. The Kaplan–Meier analysis showed the superior survival of the hemiarch and ascending aorta group compared with the total arch replacement group after long-term follow-up (76.2% vs. 68.1% vs. 64.3%, log-rank *p* < 0.001). Additionally, the logistic regression subanalysis of the groups showed a lower survival rate of the aortic arch group (OR 2.95 95% CI 1.04–7.81, *p* = 0.029). Furthermore, in our study, the more aggressive approach in the hemiarch and arch group was an independent predictor of mortality (HR = 3.446, 95% CI = 1.848–6.426, *p* < 0.001). As it was expected that the ascending aorta group would have better survival initially and that this difference would disappear afterwards, it was notable that this difference was also observed in long-term follow-up. One of the possible reasons for this is the higher number of patients at risk in the long-term follow-up of the ascending aorta group in comparison to the aortic arch group (Figure 1). Also, the rate of reoperations should be higher in patients who were only operated on their ascending aorta due to the possible extension of the dissection and the dilatation of the arch and descending aorta. However, this was not observed in our results. A possible explanation could be the fact that our centre is a major referral centre for TAAD for a large region and population and the follow-up of the majority of these patients is conducted in their regional hospitals. This potentially leads to a late referral of this patient for reoperations due to the progression of aortic dilatation or false lumen persistence and complications.

Being technically more challenging, the more extensive approach in our study had longer CPB times and circulatory arrest times. This was also noticed by Cabasa and Pochetino, who factored significantly longer aortic cross-clamp times into their results [17]. However, in our study, the aortic cross-clamp time did not differ between the groups. This can be explained by our local protocol of cross-clamping the aorta and performing proximal anastomosis first while cooling down the patient. Furthermore, Liu et al. stated that prolonged CPB time as well as prolonged circulatory arrest and aortic cross-clamp time influenced the negative outcomes [21].

A postoperative neurological deficit was present in 8–17% of patients after TAAD repair [11]. In our study, postoperative neurological deficits were more frequent in the hemiarch and arch group (17.5% vs. 6.5%). As it is more technically challenging, the more aggressive approach yielded longer circulatory arrest times as well as longer CPB times. Thus, higher complication rates, especially neurological, were expected. This was confirmed by Merkle et al. as well as Kim et al., especially for the total arch replacement subgroup. All of the authors agree that the invasiveness of the more aggressive approach and the complexity of the procedure itself, which is followed by reimplantation of the branches of the aortic arch, significantly contribute to the higher frequency of neurological events postoperatively. Yang et al. as well as Trivedi et al. offered a potential solution to this problem by supplementing the procedure with additional total carotid artery replacement [14,22]. As this procedure is technically demanding, it may be reserved for specialised high-volume aortic centres (keeping in mind that Trivedi et al. reported the results of a small cohort of patients). During long-term follow-up, we did not observe a difference between the groups in the onset of new neurological events. This fact suggests that acute postoperative events have a higher impact on outcomes. Liu et al. supported this claim, reporting that, in their cohort of patients, postoperative stroke was an independent predictor of poor outcomes [21].

The decision to choose aortic arch reconstruction is complex. Firstly, the presence of an intimal tear in this zone should be considered. Afterwards, the extension of the aortic dissections, alongside the patient’s age, comorbidities, and frailty. Also, the surgeon’s experience and centre volume and experience should be taken into account. The experience–volume relationship is especially obvious in more complex procedures like FET [4]. In the setting of TAAD, this procedure can be particularly challenging. Sun et al. reported a series of 104 patients treated with FET for TAAD. In this high-volume centre, the mortality rate was 8.4% and the false lumen was obliterated in more than 90% of patients who had a radiological follow-up in the long-term [23]. Also, the main concern in this procedure is spinal cord injury by the extension of the stent graft; special attention has to be paid to the stent length. Preventza et al. conducted a pooled analysis of 35 studies with more than 300 patients and established that spinal cord injury was much more frequent with 15 cm stents in comparison with 10 cm stents (11.6% vs. 2.5%, *p* < 0.001) [24].

Finally, in our study population, the more aggressive approach had a negative impact on both the physical and mental components of the QoL survey after long-term follow-up. This was also the conclusion of Ghazy et al., who studied 95 patients who underwent TAAD surgery. Patients who had a more extensive surgery approach reported significantly lower scores on QoL questionnaires than the ascending aorta repair group. In addition to the SF-36 questionnaire, they included the WHO-QOL-BREF questionnaire, which may have strengthened their results in favour of a more defensive strategy [25]. Future studies should focus on experience–volume both for the surgeon and the centre in adopting new complex techniques as well as an attempt to standardise the extent of the procedure for TAAD. Also, further research should facilitate the approach and decisions for less experienced surgeons and centres.

### Study Limitations

Firstly, this study is limited by its non-randomised design. However, due to the nature of the pathology, it would be extremely difficult to design a non-observational study. Secondly, the relatively small number of participants also potentially affected our findings. Thirdly, the results herein are from a high-referral-rate tertiary cardiac surgery centre that is not a dedicated aortic centre and only attending surgeons participated in the study.

## 5. Conclusions

Acute aortic dissection still remains a challenge for every cardiac surgeon. A more aggressive approach involving aortic arch repair means a lower survival rate and a lesser quality of life for patients after long-term follow-up (in comparison with the replacement of the ascending aorta). If clinically applicable, a more defensive strategy may be considered. Further investigations are needed to determine the optimal strategy for TAAD surgery, especially one that can be applied in broad clinical practice.

## Figures and Tables

**Table 1:** Baseline characteristics of patients with aortic dissection in the ascending aorta replacement group and the hemiarch and arch group.

		Ascending Aorta Replacement *n* = 108	Hemiarch and Arch *n* = 57	*p* Value
Age		58.3 ± 9.7	58.1 ± 10.4	0.906
Gender	male	87 (80.6)	42 (73.7)	0.310
	female	21 (19.4)	15 (26.3)	
Hypertension		5 (4.6)	4 (7)	0.331
Diabetes mellitus		9 (8.3)	5 (8.8)	0.521
Chronic obstructive pulmonary disease		15 (13.9)	4 (7)	0.923
Atrial fibrillation		4 (3.7)	6 (10.5)	0.189
Cardiogenic shock		5 (4.6)	2 (3.5)	0.734
Cardiac tamponade		18 (16.6)	9 (18.8)	0.857
Pericardial effusion		35(32.4)	22 (38.6)	0.238
Previous cardiac surgery		4 (2.4)	6 (5.6)	0.141
Mechanical ventilation		5 (4.6)	0 (0)	0.099
Neurological deficit (all)		15 (13.9)	10 (17.6)	0.561
Hemiparesis or hemiplegia		10 (9.2)	7 (12.2)	0.348
Coma		5 (4.6)	3 (5.3)	0.301

Values are presented as *n* (%). *p* < 0.05 was considered statistically significant.

**Table 2:** Intraoperative characteristics of patients in the ascending aorta replacement group and in the hemiarch and arch group.

		Ascending Aorta Replacement *n* = 108	Hemiarch and Arch *n* = 57	*p*
Intraoperative characteristics				
Cannulation site	Femoral artery	22 (20.3)	13 (22.8)	0.522
	Right axillary artery	17 (15.7)	10 (17.5)	
	Brachiocephalic trunk	69 (64)	34 (59.7)	
Cardiopulmonary bypass (min)		175 ± 52	241 ± 65	0.013
Aortic cross-clamp (min)		89 ± 21	98 ± 51	0.532
Circulatory arrest (min)		20 ± 7	34 ± 20	0.015
Cerebral perfusion		86 (79.6)	44 (77.2)	0.638
Bentall procedure		25 (23.1)	10 (17.5)	0.452
Coronary artery bypass grafting		6 (5.5)	3(5.7)	0.428

Data are presented as *n* (%). Values are presented as the mean ± SD. *p* < 0.05 was considered statistically significant. SD—standard deviation.

**Table 3:** Postoperative complications and follow-up characteristics of patients in the ascending aorta replacement group and in the hemiarch and arch group.

	Ascending Aorta Replacement *n* = 108	Hemiarch and Arch *n* = 57	*p*
**Postoperative complications**			
Re-exploration for bleeding	23 (21.5)	6 (10.7)	0.087
Acute kidney injury	14 (13.1)	9 (16.1)	0.603
Haemodialysis	6 (5.6)	4 (7.1)	0.698
New neurological deficit	9 (8.4)	10 (17.5)	0.045
Stroke	7 (6.5)	9 (15.7)	0.031
Coma	3 (2.8)	4 (7.1)	0.014
Pneumonia	3 (2.8)	4 (7.1)	0.194
Sepsis	21 (19.6)	12 (21.4)	0.786
Wound infectious	3 (2.8)	2 (3.5)	0.206
Lower limb ischemia	3 (2.8)	2 (3.6)	0.787
Gastrointestinal bleeding	7 (6.5)	4 (7.1)	0.885
In-hospital mortality	20 (18.5)	16 (28.1)	0.035
**Follow up characteristics**			
Reoperation	10 (9.2)	4 (7.1)	0.354
Cardiovascular event	10 (13.2)	8 (25)	0.132
Stroke	2 (2.6)	1 (3.1)	0.887

Values are presented as *n* (%). *p* < 0.05 was considered statistically significant.

**Table 4:** Cox regression analysis of the predictors for mortality.

	B	HR	95.0% CI for Exp(B)		*p* Value
			Lower	Upper	
Age	0.087	1.090	1.052	1.131	<0.001
Coronary artery disease	1.847	6.340	2.851	14.095	<0.001
Hemiarch and arch group	1.237	3.446	1.848	6.426	<0.001
Re-exploration for bleeding	0.790	2.204	1.011	4.807	0.047
Acute kidney injury	1.011	2.749	1.153	6.552	0.023
Haemodialysis	1.511	4.530	1.377	14.898	0.013
Paresis	0.943	2.568	1.005	6.560	0.049
Stroke	1.278	3.590	1.646	7.829	0.001
Coma	2.786	16.214	5.252	50.058	<0.001
Pneumonia	1.663	5.277	2.054	13.561	0.001
Sepsis	0.716	2.046	1.043	4.013	0.037

## Data Availability

Data available on reasonable request.

## References

[1] David T.E. (2015). Surgery for acute type A aortic dissection. J. Thorac. Cardiovasc. Surg..

[2] Rathore K., Newman M. (2022). Aortic Root and Distal Arch Management During Type A Aortic Dissection Repair: Expanding Horizons. Braz. J. Cardiovasc. Surg..

[3] Poon S.S., Theologou T., Harrington D., Kuduvalli M., Oo A., Field M. (2016). Hemiarch versus total aortic arch replacement in acute type A dissection: A systematic review and meta-analysis. Ann. Cardiothorac. Surg..

[4] Henn M.C., Moon M.R. (2021). Limited versus extended repair for type A aortic dissection involving the aortic arch. J. Card. Surg..

[5] Waterford S.D., Gardner R.L., Moon M.R. (2018). Extent of Aortic Replacement in Type A Dissection: Current Answers for an Endless Debate. Ann. Thorac. Surg..

[6] Chivasso P., Mastrogiovanni G., Miele M., Bruno V.D., Rosciano A., Montella A.P., Triggiani D., Colombino M., Cafarelli F., Leone R. (2021). Frozen Elephant Trunk Technique in Acute Type A Aortic Dissection: Is It for All?. Medicina.

[7] Roselli E.E., Rafael A., Soltesz E.G., Canale L., Lytle B.W. (2013). Simplified frozen elephant trunk repair for acute DeBakey type I dissection. J. Thorac. Cardiovasc. Surg..

[8] Berretta P., Patel H.J., Gleason T.G., Sundt T.M., Myrmel T., Desai N., Korach A., Panza A., Bavaria J., Khoynezhad A. (2016). IRAD experience on surgical type A acute dissection patients: Results and predictors of mortality. Ann. Cardiothorac. Surg..

[9] Bachet J. (2021). Commentary: Acute type A dissection-Should we systematically replace the aortic root?. J. Thorac. Cardiovasc. Surg..

[10] Erbel R., Aboyans V., Boileau C., Bossone E., Di Bartolomeo R., Eggebrecht H., Evangelista A., Falk V., Frank H., Gaemperli O. (2014). 2014 ESC Guidelines on the diagnosis and treatment of aortic diseases. Kardiol. Pol..

[11] Dumfarth J., Kofler M., Stastny L., Plaikner M., Krapf C., Semsroth S., Grimm M. (2018). Stroke after emergent surgery for acute type A aortic dissection: Predictors, outcome and neurological recovery. Eur. J. Cardiothorac. Surg..

[12] Pocock S.J., Clayton T.C., Altman D.G. (2002). Survival plots of time-to-event outcomes in clinical trials: Good practice and pitfalls. Lancet.

[13] Grau J.B., Kuschner C.E., Ferrari G., Wilson S.R., Brizzio M.E., Zapolanski A., Yallowitz J., Shaw R.E. (2015). Effects of a protocol-based management of type A aortic dissections. J. Surg. Res..

[14] Yang B., Norton E.L., Shih T., Farhat L., Wu X., Hornsby W.E., Kim K.M., Patel H.J., Deeb G.M. (2019). Late outcomes of strategic arch resection in acute type A aortic dissection. J. Thorac. Cardiovasc. Surg..

[15] Rylski B., Hahn N., Beyersdorf F., Kondov S., Wolkewitz M., Blanke P., Plonek T., Czerny M., Siepe M. (2017). Fate of the dissected aortic arch after ascending replacement in type A aortic dissectiondagger. Eur. J. Cardiothorac. Surg..

[16] Zierer A., Voeller R.K., Hill K.E., Kouchoukos N.T., Damiano R.J., Moon M.R. (2007). Aortic enlargement and late reoperation after repair of acute type A aortic dissection. Ann. Thorac. Surg..

[17] Cabasa A., Pochettino A. (2016). Surgical management and outcomes of type A dissection-the Mayo Clinic experience. Ann. Cardiothorac. Surg..

[18] Larsen M., Trimarchi S., Patel H.J., Di Eusanio M., Greason K.L., Peterson M.D., Fattori R., Hutchison S., Desai N.D., Korach A. (2017). Extended versus limited arch replacement in acute Type A aortic dissection. Eur. J. Cardiothorac. Surg..

[19] Easo J., Weigang E., Holzl P.P., Horst M., Hoffmann I., Blettner M., Dapunt O.E. (2013). Influence of operative strategy for the aortic arch in DeBakey type I aortic dissection—Analysis of the German Registry for Acute Aortic Dissection type A (GERAADA). Ann. Cardiothorac. Surg..

[20] Merkle J., Sabashnikov A., Deppe A.C., Zeriouh M., Maier J., Weber C., Eghbalzadeh K., Schlachtenberger G., Shostak O., Djordjevic I. (2018). Impact of ascending aortic, hemiarch and arch repair on early and long-term outcomes in patients with Stanford A acute aortic dissection. Ther. Adv. Cardiovasc. Dis..

[21] Liu H., Chang Q., Zhang H., Yu C. (2017). Predictors of Adverse Outcome and Transient Neurological Dysfunction Following Aortic Arch Replacement in 626 Consecutive Patients in China. Heart Lung Circ..

[22] Trivedi D., Navid F., Balzer J.R., Joshi R., Lacomis J.M., Jovin T.G., Althouse A.D., Gleason T.G. (2016). Aggressive Aortic Arch and Carotid Replacement Strategy for Type A Aortic Dissection Improves Neurologic Outcomes. Ann. Thorac. Surg..

[23] Ma W.G., Zhang W., Wang L.F., Zheng J., Ziganshin B.A., Charilaou P., Pan X.D., Liu Y.M., Zhu J.M., Chang Q. (2016). Type A aortic dissection with arch entry tear: Surgical experience in 104 patients over a 12-year period. J. Thorac. Cardiovasc. Surg..

[24] Preventza O., Liao J.L., Olive J.K., Simpson K., Critsinelis A.C., Price M.D., Galati M., Cornwell L.D., Orozco-Sevilla V., Omer S. (2020). Neurologic complications after the frozen elephant trunk procedure: A meta-analysis of more than 3000 patients. J. Thorac. Cardiovasc. Surg..

[25] Ghazy T., Eraqi M., Mahlmann A., Hegelmann H., Matschke K., Kappert U., Weiss N. (2017). Quality of Life after Surgery for Stanford Type A Aortic Dissection: Influences of Different Operative Strategies. Heart Surg. Forum.

